# Considerations on combination therapies for disease modification in Parkinson’s

**DOI:** 10.1038/s41531-026-01483-9

**Published:** 2026-07-17

**Authors:** Rachel M. Hughes, Aleksandra Pilcicka, Trevor Klee, Fiona Ducotterd, Heather Mortiboys, Simon R. W. Stott

**Affiliations:** 1 Cure Parkinson’s, London, UK; 2 Highway Pharmaceuticals, Boston, MA USA; 3 Alzheimer’s Research UK UCL Drug Discovery Institute, University College London, London, UK; 4 Sheffield Institute for Translational Neuroscience (SITraN), School of Medicine and Population Health, University of Sheffield, Sheffield, UK

**Keywords:** Diseases, Medical research, Neurology, Neuroscience

## Abstract

Clinical trials of monotherapies have failed to deliver disease modification for patients living with Parkinson’s disease, suggesting that a broader strategy for therapeutic development may be required. The medical research charity Cure Parkinson’s convened a workshop to discuss key considerations for developing rationally-designed combination therapies to slow progression of the condition. Here, we review combination therapies and share the conclusions of the meeting.

## Introduction

While a broad range of treatments are available for symptomatic management of Parkinson’s disease (PD), there are currently no disease-modifying therapies (DMTs) – agents that slow, stop or reverse disease progression. Lack of success in clinical trials exploring potential DMTs is likely attributable to the complexity of PD, our understanding of the causal biology, variability between patients and biomarkers for stratification, advanced stage of pathology at diagnosis, and appropriateness and sensitivity of clinical endpoints^1–3^. In addition, the majority of potential DMTs tested to date in PD trials have been individual molecules directed towards specific molecular targets or biological pathways associated with the condition and may not address enough of the complex mechanisms to impact disease progression. Combinations of treatments are increasingly being employed in other multifaceted diseases to address molecular complexity^4^. Since combining chemotherapies for the treatment of acute leukaemia in 1965^5^, combination therapies have become a “cornerstone of cancer therapy^6^”. Between January 2011 and December 2023, Chen et al. (2024) reported that of the 292 US Food and Drug Administration (FDA) approval entries (encompassing 110 drugs for oncological indications), one third were combinations^7^. Combinatorial approaches have also been successfully applied to antibacterial and antiviral interventions to address the multifactorial nature of the associated diseases^8^. There have been attempts at testing combinations of therapies in PD, from the DATATOP study in the 1990s^9^ (involving the testing of deprenyl and tocopherol) to the more recent Australian Parkinson’s Mission Study 2, evaluating the effect of ambroxol and doxycycline both separately and in combination^10^. While strategies for combination therapies are being proposed for neurodegenerative conditions^11^, the majority of clinical studies of DMTs for PD have focused on monotherapies.

Over the last decade, 84 Phase 2 and 3 DMT studies were registered on ClinicalTrials.gov, however, only three of these trials were assessing combinations of agents as potential DMTs for PD (not including herbal formulations). These have been two Phase 2 studies, assessing the combination of insulin and glutathione (NCT05266417, registered February 2023) and metabolic cofactor supplementation (L-serine, *N*-acetyl-L-cysteine, nicotinamide riboside, and L-carnitine tartrate - NCT04044131, registered July 2019)^12^, and one Phase 3 study (NCT05576818, registered October 2022) investigating the synbiotic mixture of a probiotic with prebiotic fibres^13^.

Trial evidence to date suggests it may be overly optimistic to expect a single molecule targeting a specific biological pathway to result in a clinically meaningful response across a PD patient cohort. Therefore, an alternative strategy could involve combining therapies that have evidence-based support for more than a single target or pathway, which in combination may help to expedite the development of treatments that slow this complex and heterogeneous condition^11^.

In the absence of an approved DMT, combination therapies seeking to slow PD progression represent challenging subject matter. They raise complex questions and decisions from preclinical models and clinical trial design through to regulatory implications. To address these matters and explore how best to approach combination therapies for PD, Cure Parkinson’s held a workshop in June 2025, attended by individuals from the community. The goal was to use the information gained as guidance for applicants and evaluators in future research funding calls, and as a way to stimulate discussion and inspire researchers to consider new strategies for experimental combination DMTs for PD.

## Combination drugs vs combination therapies

To begin, attendees discussed different forms of combinatorial approaches, beginning with differences between combination drugs and therapies. Combination drugs are defined as fixed-dose treatments containing two or more active pharmaceutical ingredients, an example being carbidopa/levodopa. This ‘gold standard’ symptomatic treatment for PD is a combination of two active agents that are necessary for the treatment to have the desired effect. Oral administration of the amino acid precursor levodopa alone results in the majority of the agent being converted to dopamine in the gut, leading to nausea and vomiting. It is only in combination with carbidopa (a peripheral DOPA decarboxylase inhibitor) that these side effects are reduced and brain-specific dopamine is produced at the required levels^14^. While combination drugs are often easier to administer, they lack a personalised approach due to fixed dosages.

In contrast, combination therapies involve multiple interventions administered separately – either in parallel or sequentially. Since the first joint use of streptomycin and para-aminosalicylic acid for the treatment of tuberculosis in the 1950s^15^, combination therapies have been widely applied in the oncology and antibiotic fields, where enhanced synergistic effectiveness can broaden the therapeutic spectrum and reduce the chance of resistance developing.

Pharmacologically, combination therapies can be divided into different basic subtypes - congruous, syncretic, and coalistically additive^8,16^. Congruous combinations involve agents targeting distinct but complementary, essential biological pathways or targets. As monotherapies, these agents may elicit limited efficacy; however, when administered together, they produce additive benefit. Syncretic combinations lead to a synergistic effect by simultaneously impacting essential and non-essential targets (carbidopa/levodopa is an example). Coalistically additive combinations involve agents that act on synthetically functional, non-essential targets. In this scenario, therapeutic effects are observed only by acting on both targets. Hypothetical examples of possible congruous, syncretic and coalistically additive combinations for DMTs in PD are described in a recent commentary^17^.

Combination therapies do not have to solely rely on drug-drug paradigms; drug and device or drug with lifestyle interventions may also be considered, fitting within the same three basic subtypes. A recent example of this format was a pilot study investigating the combinatorial impact of D-beta-hydroxybutyrate and nicotinamide riboside with or without exercise on brain energy metabolism (NCT04322461, registered March 2020).

Importantly, it is possible that monotherapies already tested in PD clinical trials could become components of a combination therapy. There are instances in which Phase 2 results offered encouraging initial signs of efficacy, only to be followed by disappointing Phase 3 data, for example with the exenatide trials^18,19^. Incorporating a ‘failed’ drug into a combination could potentially provide that agent with improved pharmacokinetic properties (eg. improved CNS penetrance or lower dosage with the same pharmacodynamics) via a syncretic combination, or a broader impact in a congruous combination that addresses additional biological pathways (for example, the development of CNS-penetrant dual GLP-1/GIP receptor agonists like KP405 (NCT06189170) may have greater potential as a DMT for PD than exenatide alone).

Since 2020, approximately 250 new medications have been approved by the US FDA^20^. Of these, two were combination therapies developed by the same company, Axsome Therapeutics. One of these approved treatments, “Auvelity” (dextromethorphan-bupropion), was presented and reviewed at the workshop to better understand the requirements of developing a combination drug. The first step in that process involved elucidating the mechanistic advantage of combining the agents.

## Identify the mechanisms and rationalise the combination strategy

It is important that investigators understand the individual contribution of each component within a combination therapy and why the mix of agents provides synergistic benefit. Here, Auvelity represented a useful case study for the workshop. Dextromethorphan, first described in 1946 and approved in 1958, is an over-the-counter antitussive with multiple mechanisms of action, including NMDA receptor antagonism, σ1 receptor agonism, and serotonin–norepinephrine reuptake inhibition. In addition to its cough suppressant properties, dextromethorphan also shares pharmacological properties with antidepressants^21^. However, the therapeutic benefits of dextromethorphan in this role have been limited due to rapid metabolism by CYP2D6, resulting in a half-life of 4 hours^22^. Auvelity’s second active component is bupropion, an atypical antidepressant that acts via norepinephrine and dopamine reuptake inhibition; it is also a potent CYP2D6 inhibitor^23^. Clinical testing demonstrated that, by blocking CYP2D6, bupropion is able to increase concentrations of dextromethorphan in the blood, providing a more rapid and sustained antidepressant effect than each agent alone^24^. A large number of Phase 1 clinical trials were undertaken to develop this combination. Of the 14 studies registered on ClinicalTrial.gov for the testing of Auvelity, 11 were Phase 1 trials. Despite being well-characterised generic agents, the safety, tolerability, dose optimisation, drug-food interactions, and pharmacokinetics of combining dextromethorphan and bupropion all needed to be thoroughly addressed. Following successful Phase 3 testing, Auvelity was approved by the US FDA for use as a treatment of major depressive disorder in August 2022.

For disease modification in PD, improved pharmacokinetics may increase the performance of an agent for which encouraging data have already been generated, but a more compelling case could be made for congruous combinations. A real-world example, not discussed at the workshop, is the development of AMX0035 (also known as Relyvrio) by Amylyx. This is a combination of sodium phenylbutyrate and tauroursodeoxycholic acid (TUDCA) that simultaneously targets two major pathways of neurodegeneration. Sodium phenylbutyrate acts as a chemical chaperone, inhibiting endoplasmic reticulum stress responses and neurodegeneration induced by accumulation of misfolded or mutant proteins^25^, while TUDCA inhibits mitochondrial-mediated apoptosis and formation of reactive oxygen species^26^. Both of these pathways are implicated in neurodegenerative conditions, so AMX0035 represented a congruous combination with potentially broad application. Preclinically, the combination changed more genes and metabolites than the individual components in ALS patient-derived fibroblasts^27^, and demonstrated neuroprotection in a middle cerebral artery occlusion rodent model^28^. The combination of sodium phenylbutyrate and TUDCA has also been tested in models of PD^29^. However, despite encouraging results in a Phase 2 trial for ALS^30^, subsequent Phase 3 testing could not replicate the findings^31^. While not as successful as Auvelity, AMX0035 represents a useful case study of a unique combination therapy that others can learn from.

## The importance of being unique and protecting the combination

Repurposing of generic agents for PD is often viewed as a means of accelerating the development of novel therapies. While in theory this is correct, achieving regulatory approval and a marketed product remains extremely challenging, and repurposing of individual agents should be viewed as ‘proof-of-concept’. To strengthen chances of regulatory approval, a robust case of support from the commercial world must be made in addition to clinical success. Intellectual property represents a layer of protection and to create a combination therapy, there needs to be an argument why medical professionals would not prescribe the drugs separately. In the case of Auvelity, extended-release tablets of 45 mg dextromethorphan and 105 mg bupropion were impossible to obtain generically. This formulation made Auvelity unique and difficult to replicate.

At the workshop, it was emphasised that, in order to be patented, a combination therapy must be both new and inventive. In other words, not obvious. Different options are available when thinking about patenting combinatorial therapies. Firstly, a new combination of active ingredients leads to a new composition of matter. These patents are means of protecting novel chemical compounds or mixtures of substances that exhibit a chemical union (molecular interaction). In this instance, however, it should always be assumed that if two drugs are commonly prescribed together, they would, at some point, be combined into one pill. New formulations can also provide the basis of a patentable claim, for example incorporating doses that are not ordinarily available. Finally, new medical use claims, such as new disease indications, may be possible even if a combination is already known. Importantly, information in the public domain can affect patentability, ultimately restricting the ability to get potential treatments to patients. Researchers should therefore be encouraged to seek early advice on potentially patentable combinations.

## Engage regulators early

Given the additional complexity of combination therapies, researchers should also be instructed to engage with relevant regulators as early as possible. Here we will outline the processes used by the US FDA. The FDA considers a ‘combination product’ as being composed of any combination of a drug and a device that are physically, chemically, or otherwise combined or mixed and produced as a single entity (https://www.ecfr.gov/current/title-21/chapter-I/subchapter-A/part-3/subpart-A/section-3.2). When an application involving a ‘combination product’ is submitted to the FDA, it will be handled by the Office of Combination Products (OCP). The OCP will assign the submission/inquiry to one of the regulatory authorities based on a determination of which constituent part provides the primary mode of action (https://www.fda.gov/combination-products/about-combination-products/frequently-asked-questions-about-combination-products). Importantly, the FDA does not consider a therapy consisting solely of two or more drugs to be a “combination product” (https://www.fda.gov/regulatory-information/search-fda-guidance-documents/requesting-fda-feedback-combination-products).

For drug+drug combinations, the Center for Drug Evaluation and Research is assigned to handle inquiries and reviews. For drugs that are already approved for other disease indications but are being combined for a new indication, the primary regulatory framework is the 505(b)(2) pathway (https://www.fda.gov/regulatory-information/search-fda-guidance-documents/applications-covered-section-505b2), allowing applicants to use existing safety and efficacy data from previously approved drugs to support their application. However, this requires “bridging” studies that provide pharmacokinetic data demonstrating that the new combination does not negatively alter the safety or efficacy profiles of the original drugs. For fixed-combinations, the 21 CFR 300.50 rule requires sponsors to demonstrate the meaningful contribution of each component to the claimed therapeutic effect in addition to the safety and effectiveness in the patient population. In the case of co-development of multiple new chemical entities (https://www.fda.gov/regulatory-information/search-fda-guidance-documents/codevelopment-two-or-more-new-investigational-drugs-use-combination), there is separate guidance to assist sponsors in the development of a fixed-dose combination. The FDA acknowledges that co-development generally results in less information about the individual new investigational drugs, and as such the agency believes that co-development should be reserved for situations that meet its defined criteria.

For preclinical development, the FDA recommends that sponsors build a body of evidence supporting the biological rationale for the combination in in vivo or in vitro models relevant to the condition; these studies need to compare the activity of the individual drugs with that of the combination. It should be noted that while this manuscript has focussed on the FDA, the European Medicines Agency and other regulatory bodies have their own guidance.

## Having a plan for Parkinson’s

After considering examples of how other disease indications have approached combination therapies, workshop attendees turned their attention to ways combinatorial approaches could be employed to slow PD progression. Crucially, successful disease-modifying combination therapies in other disease indications have typically begun from the foundations of an identified DMT, an advantage that the PD field is currently lacking. In terms of preclinical studies, the attendees had lengthy discussions around model organisms and which were best suited to capture specific aspects of human disease, determining that multifaceted approaches are likely to be required utilising both in vitro assays of human IPS cell-derived cultures as well as in vivo models (preferably alpha-synuclein over-expression/preformed fibril models). In addition, greater consideration will need to be given to pharmacokinetic investigations and dose-finding studies, in parallel to determining efficacy.

Clinical trials for combination therapies will be different from standard clinical trial designs. As with the Auvelity case study, clinical testing of any combinations would require extensive dose-ranging and pharmacokinetic studies, where potential pharmacodynamic interactions as well as overlapping toxicity could also be addressed^32^. For later tests of efficacy, clinical trials for these combinations are likely to be large, investigating each drug as a monotherapy, as well as incorporating the drugs in combination and including a placebo group. This situation could represent an opportunity to be explored on the new multi-arm, multi-stage (MAMS) platform trials that are currently in development for PD^33^. Other disease indications have already explored the complexities of clinically testing combinations, and much could be learned from the findings of their investigations^34–36^.

An additional challenge faced by the PD field in the development of combination therapies is patient heterogeneity. This is a common feature across disease indications that have tackled combination therapies, from the cellular level in oncology^37,38^ to more biological network approaches^39^. This topic led workshop attendees to discuss possible patient stratification protocols and whether this might help improve outcomes in clinical trials. While genotyping is currently being employed in clinical trials investigating potential DMTs^40^, stratification of the idiopathic community remains unclear. There are ongoing efforts to redefine PD based on specific biomarkers such as alpha-synuclein seeding assays^41,42^ and aromatic L-amino acid decarboxylase (AADC)^43–45^ levels in cerebrospinal fluid, as well as inflammatory signals pointing towards different at-risk subtypes^46,47^; however, validation of these groupings in DMT clinical trials is still required. Thus, the design of a combination therapy targeting specific subtypes of PD should be carefully considered.

## Additional considerations

The aim of the workshop was not to develop a clear strategy to determine how to move the field of combinatorial therapies in PD forward, but rather to start the conversation and provoke thoughts and ideas from researchers within the community. To improve the probability of finding a DMT for PD, attendees from the workshop agreed on some initial steps that could be taken when considering a combination therapy approach for PD:

- Principal investigators should mentor early career researchers, encouraging them to consider how their drug candidate of interest could be improved. More specifically, what additional components might enhance the desired effect and how might pharmacokinetics of an agent be improved?
- Funders could include questions in their grant application forms asking the applicant if they have considered additional therapies that their agent could have synergistic effects with, particularly where the proposed investigational drug has known pharmacokinetic challenges.
- Rather than focusing solely on individual targets, CRISPR-based screening studies could evaluate which combinations of druggable targets have the potential for synergy.
- Funders should consider specific funding calls focused on developing rationally designed combination therapies.

## Conclusions

Combination therapies represent a largely unexplored avenue for disease modification in PD. It is hoped that by publishing the outcomes of this workshop, researchers within the PD field will be inspired to evaluate their work and consider whether drug combinations could be explored earlier in the drug discovery process. While this type of mindset shift may take time, our ever-growing understanding of the different biological pathways involved in PD, along with learnings from other disease indications, lend themselves to the development of a combination therapy to tackle PD from multiple molecular angles. While we remain hopeful for success in ongoing clinical trials, such as the Phase 3 testing of ambroxol, as well as the introduction of several MAMS trials, we would like to propose consideration of combination therapies as an additional approach to increase the odds in our favour for uncovering a DMT for people currently living with PD.

## Data Availability

No datasets were generated or analysed during the current study.
